# Cardiopulmonary effects of apneustic anesthesia ventilation in anesthetized pigs: a new mode of ventilation for anesthetized veterinary species

**DOI:** 10.3389/fvets.2024.1378617

**Published:** 2024-05-24

**Authors:** Alex Bukoski, John Downs, David S. Hodgson, Carolina R. Le-Bert, Robert Thomen, Lucia Flors, Lori Thombs, James Bailey

**Affiliations:** ^1^Department of Veterinary Medicine and Surgery, College of Veterinary Medicine, University of Missouri, Columbia, MO, United States; ^2^Innovative Veterinary Medicine, Ponte Vedra, FL, United States; ^3^Department of Anesthesiology, College of Medicine, University of Florida, Gainesville, FL, United States; ^4^Department of Clinical Sciences, College of Veterinary Medicine, Kansas State University, Manhattan, KS, United States; ^5^U.S. Navy Marine Mammal Program, Naval Information Warfare Center Pacific, San Diego, CA, United States; ^6^Department of Radiology, School of Medicine, University of Missouri, Columbia, MO, United States; ^7^Department of Radiology, Keck School of Medicine, University of Southern California, Los Angeles, CA, United States; ^8^Department of Statistics, College of Arts and Science, University of Missouri, Columbia, MO, United States

**Keywords:** apneustic anesthesia ventilation, conventional mechanical ventilation, porcine, anesthesia, FiO_2_

## Abstract

**Objective:**

To compare the cardiopulmonary effects of apneustic anesthesia ventilation (AAV) and conventional mechanical ventilation (CMV) in anesthetized pigs and to describe a new mode of ventilation for anesthetized veterinary species.

**Study design:**

Randomized, crossover design without washout.

**Animals:**

Twelve healthy, female white Landrace pigs.

**Methods:**

Following ketamine-midazolam premedication and anesthetic induction with propofol, the trachea was intubated, and each pig was positioned in dorsal recumbency. Anesthesia was maintained with propofol and sufentanil infusions. Pigs were instrumented and their lungs were sequentially ventilated with each mode, in random order, for 1 h according to predefined criteria [fraction of inspired oxygen (FiO_2_) = 0.21, 10 mL kg^−1^ tidal volume (V_T_), and arterial carbon dioxide tension (PaCO_2_) within 40–45 mmHg]. Cardiopulmonary data were collected at baseline, 30 and 60 min. In 8 pigs, thoracic computed tomography (CT) was performed following the 60 min time point for each mode of ventilation and images were analyzed to quantify lung aeration. The effects of ventilation mode, time, and order were analyzed using repeated measures ANOVA. Paired *t*-tests were used to compare lung aeration between modes. Significance was defined as *p* < 0.05.

**Results:**

Data from 12 pigs were analyzed. A significant effect of mode was found for heart rate, mean arterial pressure (MAP), pulmonary artery occlusion pressure, cardiac index (CI), stroke volume index, systemic vascular resistance, pulmonary vascular resistance, oxygen delivery index (DO_2_I), oxygen extraction ratio (O_2_ER), V_T_, arterial oxygen tension, arterial hemoglobin saturation, PaCO_2_, end-tidal carbon dioxide tension, alveolar dead space (V_Dalv_/V_Talv_), venous admixture (${\dot{Q}}_{s}/{\dot{Q}}_{t}$), mean airway pressure, and dynamic compliance index (C_RS_I). Order effects were also observed for some cardiovascular and respiratory variables. For the eight pigs that underwent thoracic CT, AAV resulted in significantly larger proportions of normally and hyperaerated lung while CMV resulted in larger proportions of hypoaerated and atelectatic lung.

**Conclusions:**

In dorsally recumbent anesthetized pigs, ventilated with FiO_2_ = 0.21, both modes of ventilation supported adequate oxygenation while AAV resulted in higher C_RS_I, and lower V_Dalv_/V_Talv_ and ${\dot{Q}}_{s}/{\dot{Q}}_{t}$, compared with CMV. AAV was also associated with lower MAP, CI, and DO_2_I and higher O_2_ER compared with CMV. Further investigation of AAV in anesthetized animals is warranted.

## 1 Introduction

Mechanical ventilation often is utilized in the anesthetized veterinary patient to prevent respiratory gas exchange derangements including hypoxemia and hypercapnia. Such derangements are common because general anesthesia is associated with a variety of pathophysiologic pulmonary abnormalities including decreased functional residual capacity (FRC), hypoventilation, and ventilation-perfusion (V/Q) mismatching (1). In veterinary medicine, the traditional approach to managing these gas exchange impairments has included the use of elevated fraction of inspired oxygen (FiO_2_; here defined as an FiO_2_ > 0.21 needed to maintain arterial hemoglobin saturation > 90%) in combination with some form of conventional mechanical ventilation (CMV; here defined as intermittent positive pressure ventilation in the absence of alveolar recruitment maneuvers and positive end-expiratory pressure).

Although elevated FiO_2_ readily corrects hypoxemia produced by high alveolar carbon dioxide tension and V/Q mismatch, its use may encourage the formation of absorption atelectasis (2–5). Thus, the use of FiO_2_ < 1 has been investigated, although findings are mixed as to its benefit and may be species dependent [e.g., see (6–19)]. Furthermore, the use of elevated FiO_2_ in animals that are hypoxemic secondary to abnormally high venous admixture masks, rather than treats, the underlying causes (i.e., pulmonary shunt and excessive low V/Q mismatching). Compared with elevated FiO_2_, the use of CMV directly and reliably addresses the cause of hypercapnia (i.e., alveolar hypoventilation). However, CMV alone has proven to be unreliable as a treatment for hypoxemia secondary to atelectasis and elevated venous admixture. Thus, within the CMV paradigm, additional strategies have been described including flow controlled expiration (20, 21), positive end-expiratory pressure (PEEP) and alveolar recruitment maneuvers (ARMs) [e.g., see (22–34)]. Outside the CMV paradigm, strategies such as continuous positive airway pressure (CPAP) (35–37) and apneustic anesthesia ventilation (AAV) (10) have also been described.

**Figure 1:**
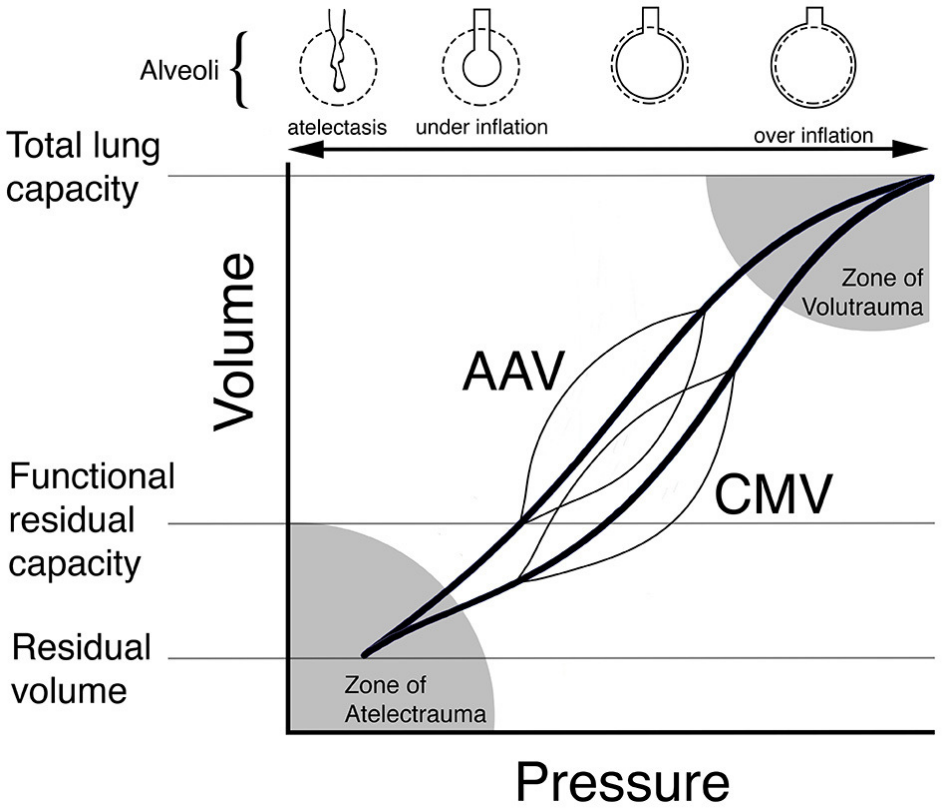
Theoretical schematic illustration of dynamic pressure-volume curves (thin lines) for conventional mechanical ventilation (CMV) and apneustic anesthesia ventilation (AAV) superimposed on individual pressure-volume curves (bold lines) for the respiratory system. AAV was designed to preserve lung architecture and compliance in the anesthetized human by periodically releasing airway pressure from a high to low level while maintaining lung volume at or above functional residual capacity (FRC). By contrast, CMV ventilates the lung from below FRC leading to the development of atelectasis, shifting of the pressure-volume curve to the right and lower compliance. In this scenario, higher pressures are required during CMV to achieve the same tidal volume. Reproduced with permission from Innovative Veterinary Medicine.

**Figure 2:**
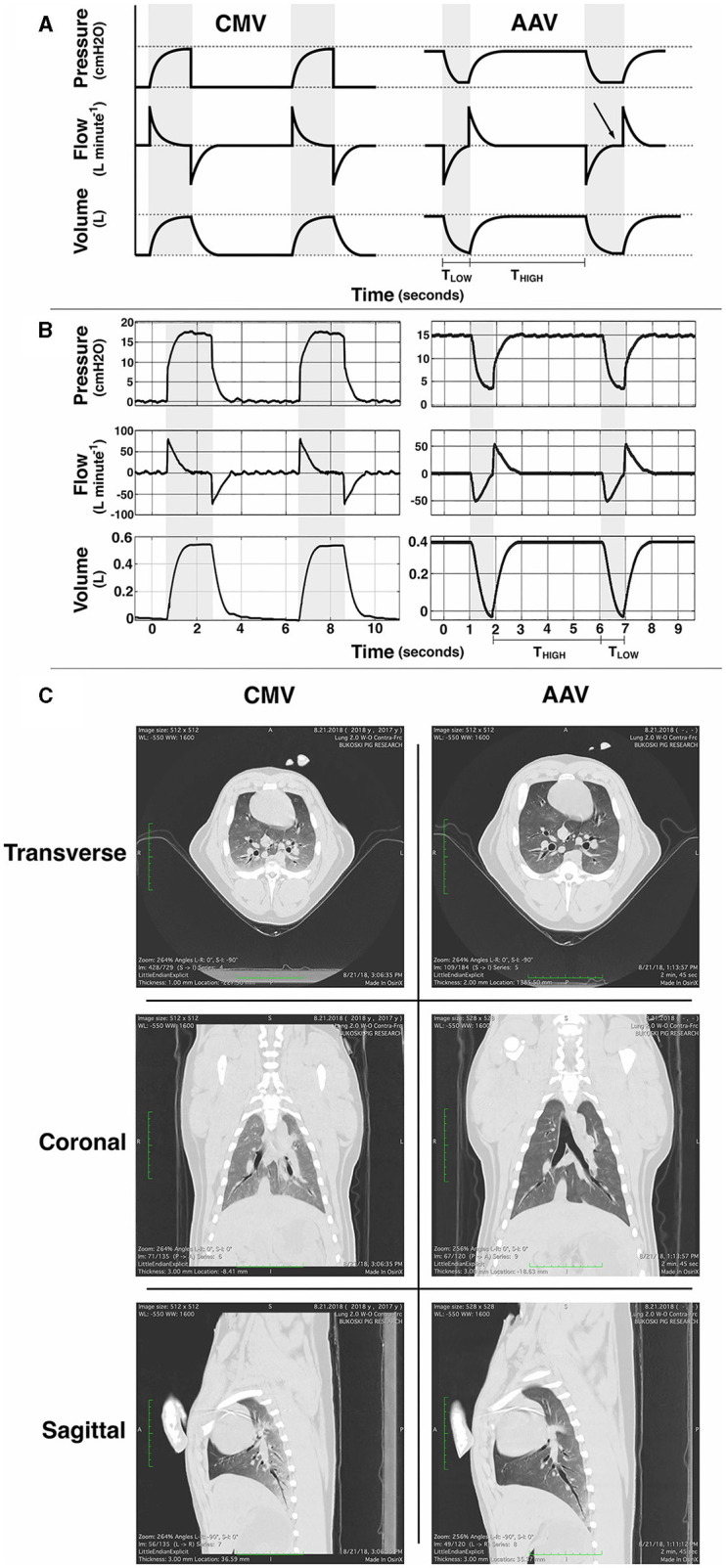
**(A)** Schematic idealized illustrations and **(B)** examples of observed pressure, flow and volume vs. time waveforms for conventional volume-controlled mechanical ventilation (CMV) and apneustic anesthesia ventilation (AAV). Gray background indicates periods of tidal ventilation corresponding to inspiratory time during CMV and the time (T_LOW_) at the low pressure setting (P_LOW_) during AAV. White background indicates expiratory time during CMV and the time (T_HIGH_) of applied high pressure (P_HIGH_) during AAV. CMV intermittently raises pressure from ambient to peak inspiratory pressure with the majority of cycle time spent at ambient pressure. AAV intermittently releases pressure from P_HIGH_ to P_LOW_ to preserve lung volume. Importantly, the duration of each AAV release (T_LOW_) is limited to prevent expiratory flow stagnation (i.e., an expiratory flow of zero). In **(A)** the first AAV release illustrates proper limitation of release time whereas the second illustrates excessively long release time with flow stagnation (arrow). **(C)** Example thoracic computed tomography (CT) images from an anesthetized pig at the end of 1 h of CMV and 1 h of AAV (with AAV as the first mode). During each scan, airway pressure was held at the lowest pressure associated with each mode for no >30 s: ambient barometric pressure for CMV and P_LOW_ for AAV. Reproduced with permission from Innovative Veterinary Medicine.

AAV is a novel mode of mechanical ventilation first described in human beings (38) and, more recently, in the horse (10). Conceptually distinct from CMV (see Figure 1) and originally described as “intermittent CPAP”, AAV is defined by 4 independent parameters: P_HIGH_, P_LOW_, T_HIGH_, and T_LOW_ (see Figure 2). The first, P_HIGH_, determines an elevated baseline pressure that is continuously applied except during time-cycled, intermittent releases to a set lower pressure (P_LOW_). These transient and rapid releases from P_HIGH_ to P_LOW_ produce tidal ventilation and carbon dioxide elimination. The time parameters T_HIGH_ and T_LOW_, which together define the duration of the respiratory cycle and determine the respiratory rate (also termed the “release rate”), are set such that T_HIGH_ (the time of applied P_HIGH_) is significantly longer than T_LOW_ (the time of applied P_LOW_). This strategy maximizes the time at P_HIGH_ which supports and promotes alveolar recruitment and maintenance of lung volume. To avoid alveolar collapse during release, T_LOW_ is set to prevent stagnation of the expiratory flow (i.e., an expiratory flow of zero) (39). At the end of each release, airway pressure is rapidly transitioned from P_LOW_ to P_HIGH_. Although designed to optimize lung function in the anesthetized human by elevating lung volume above FRC to prevent progressive formation of atelectasis and to minimize the occurrence of postoperative pulmonary complications, lung volume is rarely measured in the clinical setting. As a surrogate for lung volume, measurement of the dynamic compliance of the respiratory system via spirometry is used to set P_HIGH_ and P_LOW_ in a manner such that an optimal compliance is achieved and maintained (40). Thus, AAV represents a significant departure from the CMV paradigm in which a lower baseline pressure is intermittently cycled to a higher peak pressure to achieve tidal ventilation and most of the respiratory cycle is spent at the lower baseline pressure.

The objectives of this study were: (1) to describe an implementation of AAV in anesthetized pigs using a custom-built ventilator; and (2) to determine if this method of ventilating anesthetized pigs would provide cardiopulmonary advantages compared with traditional CMV using FiO_2_ = 0.21. We hypothesized that: (1) AAV would result in improved indices of pulmonary function (e.g., less venous admixture, less atelectasis, and higher dynamic compliance) compared with CMV; and (2) both AAV and CMV would result in acceptable indices of cardiovascular function.

## 2 Materials and methods

### 2.1 Study design

Twelve female white Landrace pigs weighing 43.1 ± 3.7 kg (mean ± standard deviation), aged between 13 and 16 weeks, and assessed to be of American Society of Anesthesiologists physical status classification 1 were each anesthetized once in a randomized, crossover design without washout to compare the cardiopulmonary effects of two modes of mechanical ventilation: apneustic anesthesia ventilation (AAV) and conventional mechanical ventilation (CMV). Experiments were performed at the University of Missouri, Veterinary Health Center and all animals were determined healthy based on limited physical examination and hematology and biochemistry profiles. Order of mode application was randomly assigned using a binary random number generator (www.random.org; Dublin, Ireland) with the constraint that six animals received AAV, and six received CMV, before transitioning to the opposite mode. At the conclusion of each experiment, animals were humanely euthanized via intravenous overdose of potassium chloride. An *a priori* power analysis (G^*^Power 3.1.9.2; University of Düsseldorf, Germany) (41) for repeated measures analysis of variance (ANOVA) design with α = 0.05, 1−β = 0.8, effect size of 0.5 and repeated measures correlation of 0.5 suggested a sample size of 10.

Each experiment proceeded in three phases: instrumentation, data collection during ventilation with the first mode, and data collection during ventilation with the second mode. The first mode of ventilation was also applied during the instrumentation phase and was initiated immediately following intubation. Each data collection period lasted 1 h, with data collected at baseline (T0), 30 min (T30), and 60 min (T60). Prior to each data collection period, a 20-min period for stabilization of monitored parameters and achievement of ventilation targets was provided. In eight animals, thoracic computed tomography (CT) imaging was performed immediately after the first and second data collection periods.

### 2.2 Anesthesia

Pigs were premedicated with intramuscular ketamine (9.14 ± 1.44 mg kg^−1^; Zetamine 100 mg mL^−1^; VETone, ID, USA) and midazolam (0.81 ± 0.15 mg kg^−1^; Midazolam Injection USP 5 mg mL^−1^; West-Ward Pharmaceuticals, NJ, USA) given in the epaxial musculature. Once the animal could be safely handled, an auricular vein was catheterized (20 GA BD Insyte; Becton Dickinson Infusion Therapy Systems Inc., UT, USA) to allow induction of general anesthesia with intravenous (IV) propofol (Diprivan 10 mg mL^−1^; Fresenius Kabi USA LLC, IL, USA) given to effect (3.0 ± 1.1 mg kg^−1^ in five animals, no propofol was required in the remaining seven) for orotracheal intubation with a cuffed endotracheal tube (Portex 8.5 mm Cuffed PVC Endotracheal Tube; Smiths Medical ASD Inc., OH, USA). Propofol infusion (Medfusion 3500 Syringe Infusion Pump; Smiths Medical ASD Inc.) was initiated at 0.5 mg kg^−1^ min^−1^ IV and, based on indicators of anesthetic depth, reduced to a maintenance level of 0.3 mg kg^−1^ min^−1^ IV for the duration of the experiment. Sufentanil (Sufentanil Citrate Injection USP 50 mcg mL^−1^; West-Ward Pharmaceuticals) was infused (Medfusion 3500 Syringe Infusion Pump; Smiths Medical ASD Inc.) at 0.05 mcg kg^−1^ min^−1^ IV throughout. A 5 mL kg^−1^ IV bolus of isotonic crystalloid (Lactated Ringer's; Baxter Healthcare, IL, USA) was followed by maintenance infusion at 5 mL kg^−1^ hr^−1^ IV by gravity drip. Body temperature was supported using an active forced-air warming device (Bair Hugger; 3M Health Care, MN, USA). Neuromuscular blocking drugs were not used.

### 2.3 Experimental protocol

Once intubated and positioned in dorsal recumbency, a spirometer (DolVent^TM^ EUI; Innovative Veterinary Medicine, FL, USA) was placed between the wye-piece of the breathing system and the tracheal tube connector for measurement of respiratory variables corrected for gas temperature and composition. Spirometer accuracy was checked at the beginning of each experimental day using a 7 L calibrated syringe (Model 4900; Hans Rudolph Inc., KS, USA) set to deliver 1 L volume to ensure measured volumes were within ±15 mL of delivered volumes. Spirometry derived variables common to both modes included tidal volume (V_T_), respiratory rate (*f*_R_), mean airway pressure (P_aw_), and total dynamic compliance of the respiratory system (C_RS_). CMV specific variables included inspiratory-to-expiratory ratio (I:E), peak inspiratory pressure (PIP) and end-expiratory pressure (EEP). AAV specific variables included P_HIGH_, P_LOW_, T_HIGH_ and T_LOW_. All respiratory variables were recorded directly from the spirometer except for airway gas concentrations (see below) and mean P_aw_ in AAV mode, which was computed offline from raw pressure-time waveforms (Matlab Version R2018b; The MathWorks Inc., MA, USA) using custom written scripts. For CMV and AAV, C_RS_ was computed as V_T_/(PIP – EEP) and V_T_/(P_HIGH_ – P_LOW_), respectively.

A multiparameter monitor (Carescape B650 with iCollect Version 5.0; GE Healthcare, Finland) was used for monitoring ECG, pulse oximetry, side-stream airway gases [FiO_2_ and end-tidal carbon dioxide tension (Pe′CO_2_)], mean systemic and mean pulmonary artery pressures (MAP and MPAP, respectively), and esophageal temperature. Airway gases were collected at the level of the machine end of the endotracheal tube. Calibration of the airway gas module with manufacturer designated gas (755571-HEL; GE Healthcare) was performed prior to each experiment. Surgical cutdown using standard techniques exposed the left carotid artery which was catheterized (4F Micro-introducer KIT-076-00; Innovative Veterinary Medicine) for systemic arterial blood sampling and pressure monitoring. Similarly, the left internal jugular vein was catheterized with an introducer (8F Catheter Introducer; Argon Medical Devices, TX, USA) through which a Swan-Ganz monitoring catheter (7F Swan-Ganz 111F7; Edwards Lifesciences LLC, CA, USA) was placed in the pulmonary artery using pressure guidance. This catheter was used for measurement of MPAP, pulmonary artery wedge-occlusion pressure (PAOP), and collection of mixed venous blood samples. The right external jugular vein was percutaneously catheterized (4F Micro-introducer KIT-076-00; Innovative Veterinary Medicine) using ultrasound guidance for injection of lithium chloride used for cardiac output determinations. For each experiment, new, disposable pressure transducers (Deltran II; Utah Medical Products Inc., UT, USA) were zeroed to atmospheric pressure and leveled to the point of the shoulder. Cardiac output was measured using lithium dilution (LiDCO Unity; LiDCO, London, UK) with the sensor connected to the carotid artery catheter. Manufacturer provided sterile lithium chloride (0.004 mmol kg^−1^ per determination) was injected using the right external jugular vein catheter according to manufacturer guidance.

At each timepoint, cardiopulmonary data were recorded, systemic arterial and mixed venous (i.e., pulmonary arterial) blood samples were simultaneously collected (safePICO 1.7 mL Aspirator; Radiometer, Brønshøj, Denmark) for immediate analysis (ABL90 Flex; Radiometer), and CO was measured. Arterial blood analysis determined arterial pH (pHa), arterial oxygen tension (PaO_2_), arterial carbon dioxide tension (PaCO_2_), and hemoglobin concentration ([Hb]). Mixed venous blood analysis determined P$\bar{\nu }$O_2_. Arterial (SaO_2_) and mixed venous (S$\bar{\nu }$O_2_) hemoglobin saturations were determined using a porcine specific oxyhemoglobin dissociation curve (42) and all blood analysis values were corrected to body temperature (43).

From this data, the following variables were calculated: stroke volume (SV) as CO/HR, alveolar dead space as a fraction of total alveolar ventilation (V_Dalv_/V_Talv_) as (PaCO_2_ – PE′CO_2_)/PaCO_2_, systemic vascular resistance (SVR) as SVR = MAP/CO, arterial oxygen content (CaO_2_) as CaO_2_ = 1.36 × [Hb] × SaO_2_ + 0.003 × PaO_2_, mixed venous oxygen content (C$\bar{\nu }$O_2_) as C$\bar{\nu }$O_2_ = 1.36 × [Hb] × S$\bar{\nu }$O_2_ + 0.00 3 × P$\bar{\nu }$O_2_, venous admixture (${\dot{Q}}_{s}/{\dot{Q}}_{t}$) as (Cc′O_2_ – CaO_2_)/(Cc′O_2_ – C$\bar{\nu }$O_2_), oxygen delivery (DO_2_) as CaO_2_ × CO, and oxygen extraction ratio (O_2_ER) as (CaO_2_ – C$\bar{\nu }$O_2_)/CaO_2_. The notation Cc′O_2_ denotes pulmonary end-capillary blood oxygen content computed from alveolar oxygen tension determined as FiO_2_ × (Pb – P_H2O_) – PaCO_2_/R – FiO_2_ × PaCO_2_ × (1 – R)/R, where R is the respiratory quotient (assumed equal to 0.8), PaCO_2_ is alveolar CO_2_ tension (approximated by PaCO_2_), Pb is barometric pressure, and P_H2O_ is the saturated vapor pressure of water. All calculations were performed at body temperature. Some variables were indexed to body mass [denoted by an appended I (e.g., SVI for stroke volume index)].

### 2.4 Mechanical ventilation

Animals were directly ventilated using medical grade air (F_I_O_2_ = 0.21) with a mechanical ventilator (DolVent^TM^, Innovative Veterinary Medicine) capable of delivering both AAV and CMV. For both modes, the V_T_ and PaCO_2_ goals were 10 mL kg^−1^ and within 40–45 mmHg, respectively. In CMV mode, the applied airway pressure and *f*_R_ were adjusted to achieve the V_T_ and PaCO_2_ goals, respectively, and the ventilator was set to maintain zero EEP. In AAV mode, ventilator settings proceeded as follows: (1) an initial *f*_R_ of 10 breaths min^−1^ was selected, (2) P_HIGH_ and P_LOW_ were adjusted up and down to achieve optimal C_RS_ while maintaining V_T_ at 10 ml kg^−1^, and (3) *f*_R_ was adjusted to meet the PaCO_2_ target. At all times during this process, T_LOW_ was set to the maximum value that prevented an expiatory flow of zero based on visual inspection of the flow waveform.

### 2.5 Computed tomography imaging

Thoracic computed tomography (CT) imaging (Aquilion 64 Fast Whole Body CT Scanner; Toshiba America Medical, CA, USA) was performed on a subset of eight randomly selected animals at the end of each 1 h ventilation period. Half of the subset received CMV as the first mode and half received AAV as the first mode. During each scan, ventilation was held at the lowest pressure associated with each mode for no >30 s: ambient barometric pressure for CMV and P_LOW_ for AAV. The scanning protocol was 120 kV, 500 mA, Eff mAs 283, FOV – Medium (320), 0.75 rotation time, HP 85, 0.5 × 64 thickness. All images were evaluated for quality by a radiologist blinded to the mode of ventilation.

Semi-automatic segmentation and quantitative analysis was performed using custom software in Matlab (Version R2018b; The MathWorks Inc.) and R (Version 4.0.5; R Foundation for Statistical Computing, Vienna, Austria). Segmentation of the lungs, major airways, and trachea from soft tissue was first performed using a semi-automatic flood-fill routine with threshold of−224 Hounsfield units (HU) and seeded within the trachea. Airway voxels were then segmented from the lungs using a similar flood-fill routine with threshold of −800 HU. A 10-pixel smoothing filter was also applied to remove airway walls. Regions of the lung parenchyma excluded from these initial segmentations such as atelectatic regions of >-224 HU were added to the lung segmentation manually using the open-source image computing platform 3D Slicer (44). The final segmented voxels were then binned into four categories by Hounsfield Unit (HU) threshold: hyperaerated (< -900 HU), normally aerated (−900 to −500 HU), hypoaerated (−500 to −100 HU), and atelectasis (>-100 HU). This allowed for quantitative analysis of absolute and relative changes in parenchymal structure across multiple ventilation methods.

### 2.6 Statistical analysis

Statistical modeling of the cardiopulmonary data utilized repeated measures three-way ANOVA with main factors mode of ventilation, time point, and order (SAS Version 9.4; SAS Institute, NC, USA). The full model, with specified covariance structure, was fit and the assumption of normality was checked for each variable by inspection of residual diagnostics including scatter plots of residuals as a function of the linear predictor, residual histograms, and residual quantile-quantile plots. These checks indicated no need for normalizing transformation. We anticipated a significant mode effect and determined the direction of the difference. For order, of interest was to compare means within mode using a reparameterized model to simplify contrast estimation. When interactions were not significant, main effects were interpreted using the overall F-statistic and *p*-value, followed by *post hoc* mean comparisons and contrast estimation using Tukey's simultaneous inference *p*-value adjustment. Paired *t*-tests were used to compare experimental times between modes of ventilation and to compare each lung inflation category defined by thoracic computed tomography imaging between modes of ventilation. Statistical significance was set at *p* < 0.05.

## 3 Results

Cardiopulmonary data from 12 pigs, and thoracic CT imaging data from 8 pigs, were analyzed. For animals receiving CMV first and AAV first, respectively, the mean ± standard deviation experimental times were: time from premedication to the start of mechanical ventilation (i.e., intubation), 34 ± 13 min and 35 ± 9 min (*p* = 0.8968); time from the start of mechanical ventilation to T0 for the first data collection period (includes instrumentation and stabilization), 86 ± 40 min and 87 ± 20 min (*p* = 0.9426); total time under the first mode of ventilation (includes instrumentation, stabilization, data collection, and time between T60 and switching to the second mode), 170 ± 33 min and 167 ± 32 min (*p* = 0.8971). Within the repeated measures ANOVA model, no statistically significant differences were found for body temperature (*p* = 0.1075). The mean ± standard deviation body temperatures for CMV and AAV, regardless of timepoint and order, were 37.3 ± 0.5 °C and 37.4 ± 0.5 °C, respectively.

Descriptive statistics for parameters unique to each ventilation mode are given in Table 1. For both modes of ventilation, these were generally stable over the 1-h data collection period. Measured and calculated respiratory and cardiovascular variables are provided in Tables 2, 3, respectively. These tables also provide *p*-values from the repeated measures ANOVA modeling that quantify the overall effect of mode (i.e., AAV vs. CMV, regardless of order or time point) and the effect of order within each mode (i.e., AAV first vs. AAV second and CMV first vs. CMV second, regardless of time point).

**Table 1:** Descriptive statistics of variables recorded during apneustic anesthesia ventilation (AAV) and conventional mechanical ventilation (CMV) with zero positive end-expiratory pressure in 12 dorsally recumbent anesthetized pigs at baseline (T0), 30 min (T30), and 60 min (T60).

**Ventilation mode**	**Variable**	**Time point**		
		**T0**	**T30**	**T60**
AAV	P_HIGH_ (cmH_2_O)	17.1 ± 1.6	16.8 ± 1.5	16.4 ± 1.7
	P_LOW_ (cmH_2_O)	4.5 ± 1.0	4.5 ± 1.1	4.4 ± 0.9
	T_HIGH_ (s)	5.1 ± 1.0	5.0 ± 1.1	4.9 ± 1.5
	T_LOW_ (s)	0.8 ± 0.1	0.8 ± 0.1	0.8 ± 0.1
CMV	PIP (cmH_2_O)	14.8 ± 2.0	14.8 ± 1.7	15.2 ± 1.7
	I:E	0.6 ± 0.1	0.6 ± 0.1	0.6 ± 0.1

Data are presented as mean ± standard deviation. P_HIGH_, high pressure; P_LOW_, low pressure; T_HIGH_, time at high pressure; T_LOW_, time at low pressure; PIP, peak inspiratory pressure; I:E, inspiratory-to-expiratory ratio.

**Table 2:** Measured and calculated respiratory variables in 12 dorsally recumbent anesthetized pigs ventilated with either apneustic anesthesia ventilation (AAV) or conventional mechanical ventilation (CMV) in random order at baseline (T0), 30 min (T30), and 60 min (T60).

**Variable**	**Order**	**AAV**			**CMV**			**Mode effect**	**Order effect**	
		**T0**	**T30**	**T60**	**T0**	**T30**	**T60**		**AAV**	**CMV**
*f*_R_ (breaths min^−1^)	AAV → CMV	10.7 ± 0.6	10.5 ± 0.6	10.5 ± 0.4	10.5 ± 0.4	10.8 ± 0.2	10.5 ± 0.2	1.0000	0.5451	0.6491
	CMV → AAV	10.0 ± 0.8	11.3 ± 1.1	11.7 ± 1.4	11.0 ± 0.4	10.8 ± 0.4	11.0 ± 0.5			
V_T_ (mL kg^−1^)	AAV → CMV	11.6 ± 0.5	11.5 ± 0.5	11.2 ± 0.5	12.7 ± 0.2	12.5 ± 0.1	12.5 ± 0.3	< 0.0001	0.6337	0.3442
	CMV → AAV	11.6 ± 0.5	11.2 ± 0.7	11.0 ± 0.7	13.4 ± 0.3	12.9 ± 0.3	12.6 ± 0.3			
PaO_2_ (mmHg)	AAV → CMV	102 ± 2	97 ± 2	95 ± 2	85 ± 2	85 ± 2	83 ± 2	< 0.0001	< 0.0001	0.3096
	CMV → AAV	89 ± 1	89 ± 2	88 ± 1	85 ± 1	83 ± 2	81 ± 1			
SaO_2_ (%)	AAV → CMV	97 ± 0	96 ± 0	96 ± 0	94 ± 0	94 ± 0	94 ± 0	< 0.0001	0.0003	0.5322
	CMV → AAV	95 ± 0	95 ± 0	95 ± 0	94 ± 0	94 ± 0	94 ± 0			
PaCO_2_ (mmHg)	AAV → CMV	39 ± 1	41 ± 1	42 ± 0	43 ±1	41 ± 0	42 ± 1	0.006	0.3667	0.1301
	CMV → AAV	40 ± 0	41 ± 1	42 ± 0	41 ± 1	41 ± 1	41 ± 1			
pHa	AAV → CMV	7.48 ± 0.01	7.47 ± 0.01	7.47 ± 0.01	7.47 ± 0.01	7.48 ± 0.01	7.48 ± 0.01	0.2465	0.1768	0.0914
	CMV → AAV	7.49 ± 0.01	7.48 ± 0.01	7.48 ± 0.01	7.49 ± 0.01	7.49 ± 0.01	7.49 ± 0.01			
PE′CO_2_ (mmHg)	AAV → CMV	40 ± 1	41 ± 1	42 ± 0	41 ± 1	40 ± 0	40 ± 1	0.0067	0.7257	0.0076
	CMV → AAV	40 ± 1	41 ± 1	42 ± 1	39 ± 1	39 ± 1	40 ± 1			
V_Dalv_/V_Talv_	AAV → CMV	−0.03 ± 0.03	0 ± 0	0 ± 0	0.04 ± 0.01	0.03 ± 0.01	0.05 ± 0.01	< 0.0001	0.0644	0.1423
	CMV → AAV	0 ± 0.01	0.02 ± 0.01	0.01 ± 0.01	0.06 ± 0.01	0.05 ± 0.01	0.04 ± 0.01			
${\dot{Q}}_{s}/{\dot{Q}}_{t}$	AAV → CMV	−0.01 ± 0.01	−0.01 ± 0	0 ± 0.01	0.04 ± 0.01	0.04 ± 0.01	0.06 ± 0.01	< 0.0001	0.0004	0.0327
	CMV → AAV	0.03 ± 0	0.03 ± 0	0.03 ± 0	0.05 ± 0.01	0.07 ± 0.01	0.08 ± 0.01			
Mean P_aw_ (cmH_2_O)	AAV → CMV	14 ± 0	14 ± 1	14 ± 1	5 ± 0	5 ± 0	5 ± 0	< 0.0001	0.8891	0.2152
	CMV → AAV	15 ± 0	14 ± 1	14 ± 1	6 ± 0	6 ± 0	6 ± 0			
C_rs_I (mL cmH_2_O^−1^ kg^−1^)	AAV → CMV	0.92 ± 0.05	0.91 ± 0.05	0.91 ± 0.06	0.95 ± 0.05	0.91 ± 0.05	0.86 ± 0.05	< 0.0001	0.7228	0.1812
	CMV → AAV	0.93 ± 0.04	0.94 ± 0.04	0.94 ± 0.03	0.84 ± 0.02	0.82 ± 0.01	0.82 ± 0.02			

For each variable, data are given for each order of mode application: AAV followed by CMV (AAV → CMV) and CMV followed by AAV (CMV → AAV).

All values are mean ± standard error of the mean. The two right-most columns provide p-values for the overall effects of mode of ventilation and order of mode application determined by data modeling with repeated measures ANOVA.

f_R_, respiratory rate; V_T_, tidal volume; PaO_2_, arterial oxygen tension; SaO_2_, arterial hemoglobin saturation; PaCO_2_, arterial carbon dioxide tension; pHa, arterial pH; PE′CO_2_, end-tidal carbon dioxide; V_Dalv_/V_Talv_, alveolar dead space; $\frac{{\dot{Q}}_{s}}{{\dot{Q}}_{t}}$, venous admixture; Mean P_aw_, mean airway pressure; C_RS_I, dynamic compliance index of the respiratory system.

**Table 3:** Measured and calculated cardiovascular variables in 12 dorsally recumbent anesthetized pigs ventilated with either apneustic anesthesia ventilation (AAV) or conventional mechanical ventilation (CMV) in random order at baseline (T0), 30 min (T30), and 60 min (T60).

**Variable**	**Order**	**AAV**			**CMV**			**Mode effect**	**Order effect**	
		**T0**	**T30**	**T60**	**T0**	**T30**	**T60**		**AAV**	**CMV**
HR (beats min^−1^)	AAV → CMV	117 ± 7	118 ± 5	118 ± 4	101 ± 4	95 ± 5	95 ± 5	< 0.0001	0.1604	0.1652
	CMV → AAV	135 ± 8	131 ± 9	127 ± 9	112 ± 11	110 ± 14	110 ± 14			
MAP (mmHg)	AAV → CMV	61 ± 3	61 ± 2	63 ± 4	70 ± 2	73 ± 3	73 ± 4	< 0.0001	0.1751	0.0027
	CMV → AAV	58 ± 2	71 ± 4	69 ± 3	83 ± 3	83 ± 3	84 ± 2			
MPAP (mmHg)	AAV → CMV	24 ± 1	24 ± 1	24 ± 1	24 ± 2	23 ± 1	24 ± 1	0.4465	0.5367	0.4646
	CMV → AAV	24 ± 2	23 ± 1	23 ± 1	23 ± 1	23 ± 1	23 ± 1			
PAOP (mmHg)	AAV → CMV	13 ± 1	13 ± 1	13 ± 1	6 ± 1	7 ± 1	7 ± 1	< 0.0001	0.5165	0.0991
	CMV → AAV	14 ± 1	13 ± 1	13 ± 1	5 ± 0	5 ± 1	5 ± 1			
CI (mL min^−1^ kg^−1^)	AAV → CMV	68 ± 4	70 ± 3	73 ± 4	106 ± 6	108 ± 7	119 ± 5	< 0.0001	0.0117	0.1499
	CMV → AAV	77 ± 4	85 ± 5	83 ± 7	106 ± 5	99 ± 3	109 ± 5			
SVI (mL beat^−1^ kg^−1^)	AAV → CMV	0.61 ± 0.06	0.58 ± 0.03	0.62 ± 0.03	1.09 ± 0.06	1.19 ± 0.07	1.31 ± 0.06	< 0.0001	0.6843	0.0009
	CMV → AAV	0.57 ± 0.02	0.65 ± 0.02	0.66 ± 0.02	0.98 ± 0.08	0.98 ± 0.12	1.06 ± 0.10			
SVR (dyne sec cm^−5^)	AAV → CMV	1,683 ± 146	1,698 ± 89	1,622 ± 115	1,226 ± 92	1,304 ± 139	1,145 ± 88	< 0.0001	0.2014	0.0440
	CMV → AAV	1,423 ± 74	1,575 ± 90	1,547 ± 55	1,499 ± 134	1,579 ± 107	1,453 ± 112			
PVR (dyne sec cm^−5^)	AAV → CMV	313 ± 24	317 ± 16	285 ± 28	322 ± 36	297 ± 23	272 ± 20	0.0002	0.0104	0.7656
	CMV → AAV	235 ± 23	210 ± 13	226 ± 20	319 ± 27	332 ± 27	294 ± 25			
DO_2_I (ml min^−1^ kg^−1^)	AAV → CMV	9.0 ± 0.7	9.2 ± 0.5	9.4 ± 0.7	13.2 ± 0.5	13.0 ± 1.1	14.2 ± 0.8	< 0.0001	0.1756	0.8828
	CMV → AAV	9.7 ± 0.6	10.4 ± 0.6	10.0 ± 0.8	13.8 ± 0.8	12.6 ± 0.4	13.8 ± 0.7			
O_2_ER	AAV → CMV	0.50 ± 0.03	0.51 ± 0.03	0.52 ± 0.04	0.37 ± 0.01	0.40 ± 0.01	0.41 ± 0.01	< 0.0001	0.4813	0.0737
	CMV → AAV	0.51 ± 0.03	0.50 ± 0.02	0.47 ± 0.02	0.33 ± 0.02	0.34 ± 0.01	0.36 ± 0.01			

All values are mean ± standard error of the mean. The two right-most columns provide p-values for the overall effects of mode of ventilation and order of mode application determined by data modeling with repeated measures ANOVA.

HR, heart rate; MAP, mean arterial pressure; MPAP, mean pulmonary artery pressure; PAOP, pulmonary artery occlusion pressure; CI, cardiac index; SVI, stroke volume index; SVR, systemic vascular resistance; PVR, pulmonary vascular resistance; DO_2_I, oxygen delivery index; O_2_ER, oxygen extraction ratio. For each variable, data are given for each order of mode application: AAV followed by CMV (AAV → CMV) and CMV followed by AAV (CMV → AAV).

No mode differences or order effects were found for *f*_R_ and spontaneous ventilation was not observed in either mode. For both modes of ventilation, V_T_ exceeded the target value of 10 mL kg^−1^ with those during CMV larger than those during AAV by approximately 1 mL kg^−1^ (*p* < 0.0001). Both PaO_2_ and SaO_2_ were higher for AAV compared with CMV (*p* < 0.0001 and *p* < 0.0001, respectively). When AAV was applied first, PaO_2_ and SaO_2_ were higher than when AAV was applied after CMV. For PaCO_2_, mode differences of ~1–2 mmHg were observed with higher values for CMV compared with AAV (*p* = 0.006), although an order effect was not observed. This contrasts with PE′CO_2_ values which were higher for AAV compared with CMV by ~1 mmHg (*p* = 0.0067). Within CMV, PE′CO_2_ was higher when CMV was applied after, rather than before, AAV. No mode or order differences were detected for pHa. Alveolar dead space and ${\dot{Q}}_{s}/{\dot{Q}}_{t}$ were lower (*p* < 0.0001 and *p* < 0.0001, respectively), and mean P_aw_ and C_RS_I were higher (*p* < 0.0001 and *p* < 0.0001, respectively), for AAV compared with CMV. Of these variables, only ${\dot{Q}}_{s}/{\dot{Q}}_{t}$ exhibited an order effect. Within AAV mode, ${\dot{Q}}_{s}/{\dot{Q}}_{t}$ was higher when AAV was applied after CMV and, within CMV mode, ${\dot{Q}}_{s}/{\dot{Q}}_{t}$ was higher when CMV was applied first.

All cardiovascular variables, except for MPAP, evidenced mode effects and/or order effects. Variables exhibiting mode effects, but not order effects, included: HR (higher for AAV, *p* < 0.0001), PAOP (higher for AAV, *p* < 0.0001), DO_2_I (lower for AAV, *p* < 0.0001), and O_2_ER (higher for AAV, *p* < 0.0001). MAP (*p* < 0.0001), CI (*p* < 0.0001), and SVI (*p* < 0.0001) were lower during AAV compared with CMV. MAP was also higher within CMV mode when CMV was applied first vs. when it was applied second. Within AAV mode, CI was higher when AAV was applied second vs. when it was applied first. However, no order effects within AAV mode were observed for SVI. Rather, for SVI, this variable was higher within CMV mode when CMV was applied first vs. when it was applied second. SVR was higher during AAV and, within CMV, it was higher when CMV was applied first. PVR was lower during AAV compared with CMV (*p* = 0.0002) and, within AAV, was lower when AAV was applied second.

Proportions of lung volume classified as hyperaerated, normally aerated, hypoaerated, and atelectatic are given in Table 4 along with the results of the paired *t*-tests used for mode comparisons within each category. Significant differences between modes were detected in all categories with AAV resulting in larger proportions of hyperaerated and normally aerated regions and smaller proportions of hypoaerated and atelectatic regions, compared with CMV. Due to the number of pigs that underwent thoracic CT imaging, it was not possible to include order of mode application as a factor in the statistical analysis.

**Table 4:** Proportions of lung volume classified as hyperaerated, normally aerated, hypoaerated, and atelectatic in eight anesthetized pigs ventilated with apneustic anesthesia ventilation (AAV) and conventional mechanical ventilation (CMV) in random order.

**Lung region**	**AAV**	**CMV**	***p*-value**
Hyperaerated	0.05 ± 0.01	0.03 ± 0.01	0.002
Normally Aerated	0.73 ± 0.02	0.61 ± 0.03	< 0.0001
Hypoaerated	0.21 ± 0.03	0.33 ± 0.04	< 0.0001
Atelectatic	0.01 ± 0.00	0.03 ± 0.01	0.0062

Paired t-tests were used to compare proportions of lung volume between modes for each lung region. Data are presented as mean ± standard error of the mean.

## 4 Discussion

This study describes a comparison of a new mode of ventilation for animals, AAV, to traditional CMV using low FiO_2_ in pigs anesthetized with propofol-based total intravenous anesthesia. Overall, statistically significant mode effects were found for many respiratory and cardiovascular variables. Notably, AAV resulted in higher PaO_2_, lower V_Dalv_/V_Talv_, lower ${\dot{Q}}_{s}/{\dot{Q}}_{t}$, and higher C_RS_I. However, AAV also resulted in lower MAP, lower CI, lower DO_2_I, and higher O_2_ER. Although less common than the effects of mode of ventilation on cardiopulmonary variables, the use of a cross-over design without washout introduced the statistical factor of order of mode application (i.e., whether AAV or CMV was applied first).

Ventilation strategies for both AAV and CMV were designed to target normocapnia (here defined as PaCO_2_ between 40 and 45 mmHg) and a standard V_T_ of 10 mL kg^−1^ to normalize these variables between modes. These choices, particularly that of normocapnia, resulted in significantly higher mean P_aw_ for AAV vs. CMV by ~8–9 cmH_2_O. Because mean airway pressure is an important determinant of venous return and cardiac output (45), the finding of reduced cardiovascular performance, particularly reduced CI and SVI and increased HR, during AAV compared to CMV was not overly surprising but was somewhat unexpected based on one of the authors (JD) clinical experience in humans. Secondary consequences of this effect were reduced DO_2_I and increased O_2_ER for AAV vs. CMV. Increased mean P_aw_ during AAV also likely contributed to higher PVR and PAOP in this group compared with the CMV group. However, no differences in MPAP were observed between modes. Higher SVR values during AAV can be largely accounted for by observed mode differences when AAV was applied before CMV. Within this ordering, SVR values decreased on switching from AAV to CMV and this change may also be related to reduced mean P_aw_ during CMV.

When AAV was compared to CMV in dorsally recumbent horses anesthetized with isoflurane, no differences in cardiovascular performance or dobutamine consumption were observed (10). However, in that study, AAV was implemented to maintain moderate hypercapnia while CMV was implemented to maintain normocapnia. The combination of this result without those of the current study, highlights the importance of intentional hypercapnia when implementing AAV. Mild to moderate hypercapnia in the anesthetized patient has been shown to result in increased levels of circulating catecholamines, improved cardiovascular function, and improved tissue oxygenation compared to maintenance of normocapnia (46–50). Further, maintenance of a moderate degree of hypercarbia causes an increase in body stores of CO_2_ that must be eliminated following recovery from anesthesia, resulting in an increase in post-anesthesia ventilation, less likelihood of post-anesthesia hypoventilation, and more rapid emergence from inhalation anesthesia (51–54). Thus, PaCO_2_ management may play an important role in determining cardiorespiratory performance during ventilation with AAV and intentional hypercapnia should be considered a fundamental component of any AAV implementation.

Another important attribute of the ventilation strategies utilized in the current study was their static nature. That is, once the predefined ventilation endpoints were reached, and following timepoint T0, additional ventilation parameter adjustment or lung recruitment were not performed. Note, however, that for AAV (but not for CMV) there was the further requirement to maximize C_RS_I, in addition to meeting both the PaCO_2_ and V_T_ goals. This choice may also have contributed to the higher mean P_aw_ observed during AAV compared to CMV. Regardless, in the authors' clinical experience, AAV is not a static mode of ventilation but is a rather dynamic mode wherein parameter adjustment is guided by feedback from both respiratory and cardiovascular monitoring. Thus, while AAV effects patient physiology it is also responsive to patient physiology and the practitioner will find that realized P_HIGH_, P_LOW_, T_HIGH_, T_LOW_, and mean P_aw_ will vary from one clinical scenario to another with the goal of balancing the beneficial respiratory effects of AAV against any observed cardiovascular impairment.

In a randomly chosen subset of 8 pigs, thoracic CT imaging was performed to assess lung aeration. We found that AAV resulted in larger proportions of hyperaerated and normally aerated lung while CMV resulted in larger proportions of hypoaerated and atelectatic lung. Typically, hyperaerated lung regions have less blood flow, resulting in high V/Q mismatch, the extreme of which is alveolar deadspace. However, alveolar deadspace was significantly lower during AAV than during CMV, indicating maintenance of an appropriate balance between alveolar ventilation and pulmonary blood flow during AAV. During CMV, there was little evidence of hyperaerated lung, yet alveolar dead space was significantly higher than observed during AAV. Even though mean Paw was lower during CMV, it appears that the intermittent increase in Paw during inhalation was sufficient to impede blood flow to normally aerated lung regions creating extreme increase in V/Q and alveolar deadspace. It is widely accepted that PE′CO_2_ rarely is an accurate reflection of PaCO_2_ during CMV. Our findings suggest that PE′CO_2_ is a better representative of PaCO_2_ during AAV. CT findings also are compatible with our calculation of ${\dot{Q}}_{s}/{\dot{Q}}_{t}$ which was higher during CMV compared to during AAV.

Although the use of elevated FiO_2_–as defined in this study—during general anesthesia is common in both veterinary and human practice, we elected to use FiO_2_ = 0.21 in this study because high FiO_2_ masks the effects of low V/Q lung regions on PaO_2_ by largely compensating for their impact on venous admixture. Thus, use of FiO_2_ = 0.21 provided a better test of each mode's ability to counteract the detrimental effects of recumbency and anesthesia on V/Q matching in the lung. Additionally, low FiO_2_ minimized the conversion of low V/Q regions to shunt via the mechanism of absorption atelectasis. It is significant that even in the absence of supplemental inspired oxygen, PaO_2_ was acceptable in all animals regardless of the mode of ventilation. In fact, if a disruption in ventilation or V/Q had occurred, monitoring with a pulse oximeter would have alerted the anesthesiologist of the disruption in pulmonary function more rapidly than if an elevated FiO_2_ was employed, allowing a rapid increase in FiO_2_, while appropriate measures to correct the abnormality are applied. The long-standing dogma that an elevated FiO_2_ provides a margin of safety is questionable (55–57).

Order effects were observed for some respiratory and cardiovascular variables. For both PaO_2_ and SaO_2_, values during AAV were higher when AAV was the first applied mode and lower when it was applied after CMV. These results are supported by the order and mode effects observed for AAV and CMV on ${\dot{Q}}_{s}/{\dot{Q}}_{t}$. While ${\dot{Q}}_{s}/{\dot{Q}}_{t}$ was lower during AAV compared to CMV, it was higher during AAV when CMV was the first applied mode. This indicates that while AAV applied after CMV tended to reduce ${\dot{Q}}_{s}/{\dot{Q}}_{t}$ and improve oxygenation, as implemented it was unable to completely reverse the larger amounts of ${\dot{Q}}_{s}/{\dot{Q}}_{t}$ that developed during ventilation with CMV. For cardiovascular variables, a significant order effect was observed for MAP during CMV with values approximately 10 mmHg higher when CMV was applied first compared to when CMV was applied after AAV. This data indicates that AAV continued to impact hemodynamics after its discontinuation and is an interesting observation that would not have been apparent were a cross-over design without washout not been used.

Limitations of this study include the use of total intravenous anesthesia, a laboratory rather than clinical setting, and the absence of a washout period between treatments. Total intravenous anesthesia and inhalational anesthesia may result in differential effects on cardiopulmonary performance which were not assessed in the current study. This is deserving of additional research as inhalational anesthetics are more commonly used in veterinary practice than are total intravenous techniques. While controlled laboratory studies are necessary to establish baseline effects of new ventilatory strategies, it is the impact of such strategies in a diverse clinical population that determines their ultimate utility. Thus, investigations of AAV in such populations, and in other species, are warranted. Although the absence of a washout period did result in some novel observations (see above), the inclusion of a washout period would have likely removed the confounding effect of order of mode application and resulted in a clearer picture of each mode's cardiopulmonary effects. Lastly, it may be argued that the addition of PEEP would improve gas exchange during CMV. CMV with PEEP has been compared to airway pressure release ventilation (APRV; a mode closely related to AAV) in dogs (58). That study found that, compared with CMV+PEEP, APRV resulted in enhanced alveolar ventilation without compromise of circulatory function and tissue oxygen balance.

In conclusion, we have described the cardiopulmonary effects of AAV which is a novel mode of mechanical ventilation in veterinary anesthesia. AAV as implemented in this study resulted in improved indices of pulmonary performance (notably higher C_RS_I, and lower V_Dalv_/V_Talv_ and ${\dot{Q}}_{s}/{\dot{Q}}_{t}$) compared with CMV in dorsally recumbent anesthetized pigs ventilated with FiO_2_ = 0.21. However, the cardiovascular impact of AAV was greater than CMV with AAV associated with lower MAP, CI, and DO_2_I and higher O_2_ER compared to CMV. Importantly, based on the established beneficial cardiovascular effects of intentional hypercapnia during anesthesia (46–49), this comparative negative effect of AAV (compared with CMV) on cardiovascular performance may be mitigated by judicious use of intentional hypercapnia during anesthetic management. Further investigation of AAV in anesthetized animals, particularly in clinical settings and with intentional hypercapnia, is warranted.

## Data availability statement

The raw data supporting the conclusions of this article will be made available by the authors, without undue reservation.

## Ethics statement

The animal study was approved by the University of Missouri, Animal Care and Use Committee and the Department of the Navy, Bureau of Medicine and Surgery. The study was conducted in accordance with the local legislation and institutional requirements.

## Author contributions

AB: Conceptualization, Data curation, Funding acquisition, Investigation, Methodology, Project administration, Validation, Writing – original draft, Writing – review & editing. JD: Investigation, Methodology, Writing – original draft, Writing – review & editing. DH: Investigation, Methodology, Writing – original draft, Writing – review & editing. CL-B: Investigation, Methodology, Writing – original draft, Writing – review & editing. RT: Formal analysis, Methodology, Software, Writing – original draft, Writing – review & editing. LF: Formal analysis, Methodology, Writing – original draft, Writing – review & editing. LT: Formal analysis, Writing – original draft, Writing – review & editing. JB: Conceptualization, Data curation, Funding acquisition, Investigation, Methodology, Project administration, Validation, Visualization, Writing – original draft, Writing – review & editing.
